# Urocolpos et reflux d'urine dans l'utérus dans les suites de mutilations génitales féminines au Centre hospitalier universitaire régional de Ouahigouya, Burkina Faso

**DOI:** 10.48327/MTSI.2021.186

**Published:** 2021-12-03

**Authors:** Sansan Rodrigue SIB, Évelyne KOMBOIGO, Moussa SANOGO, Issa OUÉDRAOGO, Sibraogo KIEMTORE, Ali OUÉDRAOGO

**Affiliations:** 1 Service de gynécologie obstétrique au CHU régional de Ouahigouya, Burkina Faso; 2 Département de gynécologie obstétrique et de médecine de la reproduction, CHU Sourou Sanon de Bobo Dioulasso, Burkina Faso; 3 Département de gynécologie obstétrique au CHU Yalgado Ouédraogo, Ouagadougou, Burkina Faso; 4 Département de gynécologie obstétrique au CHU de Tengandogo, Ouagadougou, Burkina Faso

**Keywords:** Mutilation génitale féminine, Infibulation, Urocolpos, Rétention d'urine, Reflux intra-utérin d'urine, Hôpital, Ouahigouya, Burkina Faso, Afrique subsaharienne, Female genital mutilation, Infibulation, Urocolpos, Urinary retention, Intrauterine urine reflux, Hospital, Ouahigouya, Burkina Faso, Sub-Saharan Africa

## Abstract

**Introduction:**

L'urocolpos est rare, de même que le reflux d'urine dans l'utérus qui peut lui être associé. Les mutilations génitales féminines (MGF) en sont des causes rarement décrites. Nous en présentons deux cas dont l'un est associé à un reflux d'urine dans l'utérus.

**Cas cliniques:**

Deux fillettes de 7 ans et de 15 mois ont présenté dans les suites de MGF, des douleurs pelviennes, une dysurie de poussée et des épisodes de rétention d'urine.

**Résultats:**

C'est l’échographie pelvienne qui a permis d'objectiver un urocolpos associé chez la patiente de 7 ans à un reflux d'urine dans la cavité utérine. La bactériologie a isolé *Escherichia coli* des urines. La désinfibulation et une antibiothérapie ont rétabli une miction normale. L'urocolpos et le reflux d'urine dans l'utérus peuvent être dus aux MGF.

**Conclusion:**

Certains signes peuvent faire penser à un urocolpos dans le contexte de MGF, mais l’échographie est importante pour le diagnostic.

## Abstract (en)

**Introduction:**

Urocolpos is rare as is the reflux of urine into the uterus which may be associated with it. Female genital mutilation (FGM) is a rarely described cause. We present two cases, one of which is associated with reflux of urine into the uterus.

**Clinical cases:**

Two 7-year-old and 15-month-old girls presented after FGM with pelvic pain, pushing dysuria, and episodes of urine retention.

**Results:**

It was the pelvic ultrasound which made it possible to objectify an urocolpos associated in the 7-year-old patient with reflux of urine into the uterine cavity. Bacteriology has isolated *Escherichia coli* from urine. Deinfibulation and antibiotic therapy restored normal urination. Urocolpos and reflux of urine into the uterus may be due to FGM.

**Conclusion:**

There are some signs that suggest urocolpos in the context of FGM, but the ultrasound is important for the diagnosis.

## Introduction

L'urocolpos est une distension du vagin due à un épanchement d'urine lors de la miction. Il a été le plus souvent décrit lors de la coalescence des petites lèvres survenant en dehors des mutilations génitales féminines [4]. Celles-ci sont une cause non décrite d'urocolpos. Nous présentons deux cas d'urocolpos infectés survenus dans les suites de mutilations génitales et pris en charge au Centre hospitalier universitaire régional de Ouahigouya au Burkina Faso. L'un des deux cas est associé à un reflux d'urine dans l'utérus. Notre objectif est de sensibiliser sur la possibilité de cette complication, étant donné que les mutilations génitales féminines sont encore fréquentes dans de nombreuses régions du monde.

## Cas cliniques

### Cas clinique 1

Une fillette de 7 ans a été reçue en consultation pour des douleurs pelviennes, une dysurie de poussée et des épisodes de rétention d'urine. Les symptômes évoluaient de façon intermittente depuis environ une année, après que l'enfant a été victime d'une mutilation génitale rituelle. La patiente a été amenée plusieurs fois en consultation dans le centre de santé de son village où de l'amoxicilline et du paracétamol lui auraient été souvent prescrits. Elle aurait été également référée à maintes reprises au Centre hospitalier universitaire régional de Ouahigouya pour une meilleure prise en charge, mais ses parents ne l'y avaient pas amenée. C'est la persistance des signes qui les aurait persuadés d'accepter plus tard la référence.

**Figure 1:**
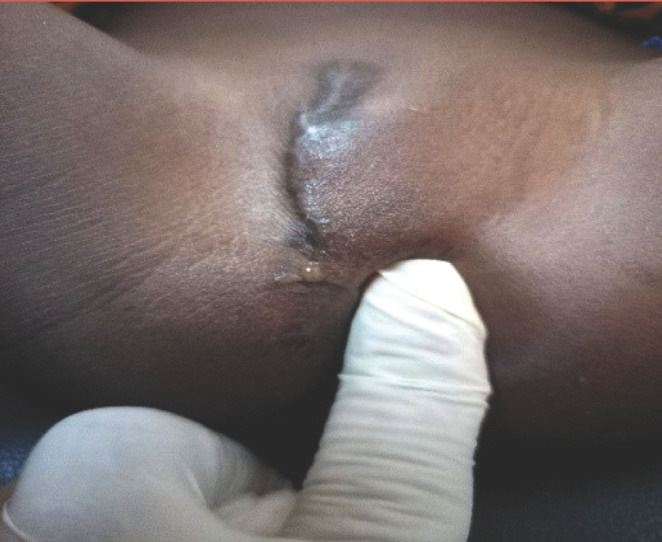
Infibulation avec excision asymétrique et accolement des grandes lèvres. La pression de la vulve laisse s’écouler une goutte d'urine au niveau du pertuis Infibulation with asymmetric excision and adhesion of the labia majora. The pressure of the vulva lets a drop of urine flow through the small hole

L'examen physique a retrouvé un état général conservé, une absence de syndrome infectieux, une légère voussure hypogastrique et une masse hypogastrique rénitente, douloureuse et mate. Au niveau de la vulve, il existait une cicatrice fibreuse d'une infibulation, avec accolement des grandes lèvres. Le siège de la cicatrice témoignait d'une excision large et asymétrique des grandes lèvres. Il existait un petit pertuis au sein de la fibrose, repérable seulement lors de la miction ou lorsque l'on exerçait une pression sur l'hypogastre ou sur la vulve qui était rénitente en regard de l'orifice vaginal fermé (Fig. 1). Le sondage vésical était donc impossible. Le diagnostic présomptif d'une infibulation compliquée de sténose du méat urétral et d’épisodes de rétention aiguë d'urine a été posé.

**Figure 2:**
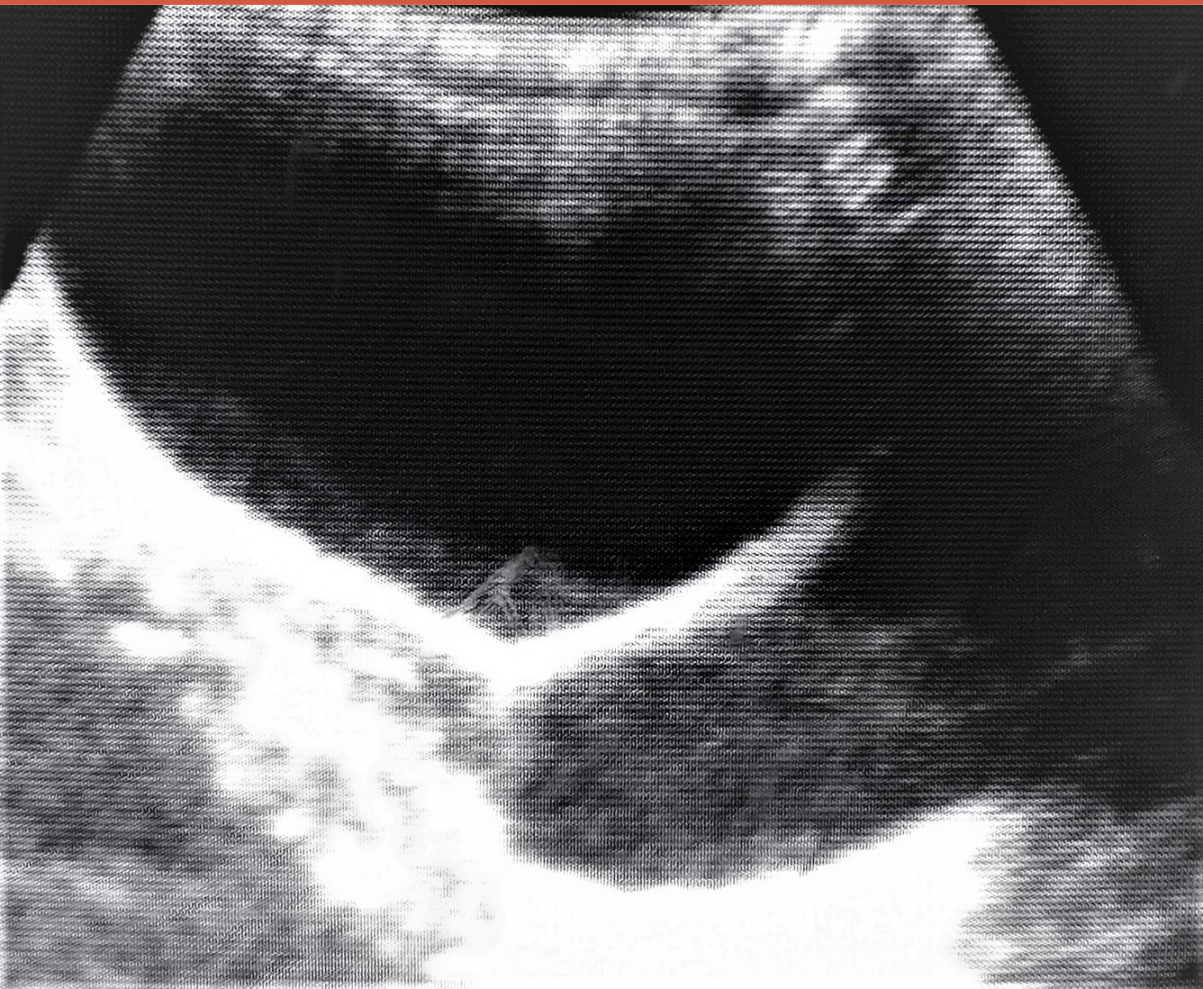
Image échographique avant la miction, montrant une réplétion vésicale et une autre image liquidienne Ultrasound image obtained before voiding, showing full bladder and another fluid image

**Figure 3:**
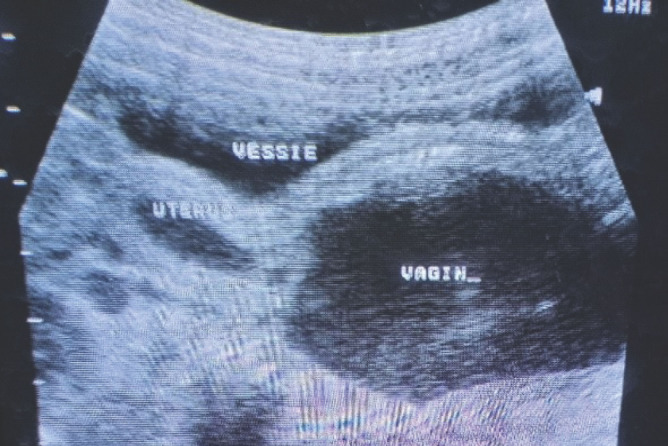
Image échographique post-mictionnelle montrant un urocolpos et un épanchement dans la cavité utérine Post voiding ultrasound picture showing urocolpos and fluid effusion in the uterine cavity

L’échographie abdomino-pelvienne a été réalisée en urgence. Dans un premier temps le passage de la sonde était douloureux, entraînant une agitation de la patiente. De ce fait, l'exploration précise des organes intra-abdominaux n'a pas été possible. Cependant une distension vésicale avec un volume urinaire de 193 ml et une autre image liquidienne ont été visualisées (Fig. 2). L'examen a été suspendu pour permettre à la patiente d'uriner. Après une miction difficile et longue, l’échographie a été reprise. Elle était non douloureuse dans ce second temps, et a permis de noter un résidu post-mictionnel de 41 ml, ainsi qu'un épanchement liquidien dans le vagin et l'utérus (Fig. 3). Il n'y avait pas de dilatation des cavités rénales. Sur le plan biologique, la créatininémie et l'hémogramme étaient normaux.

**Figure 4:**
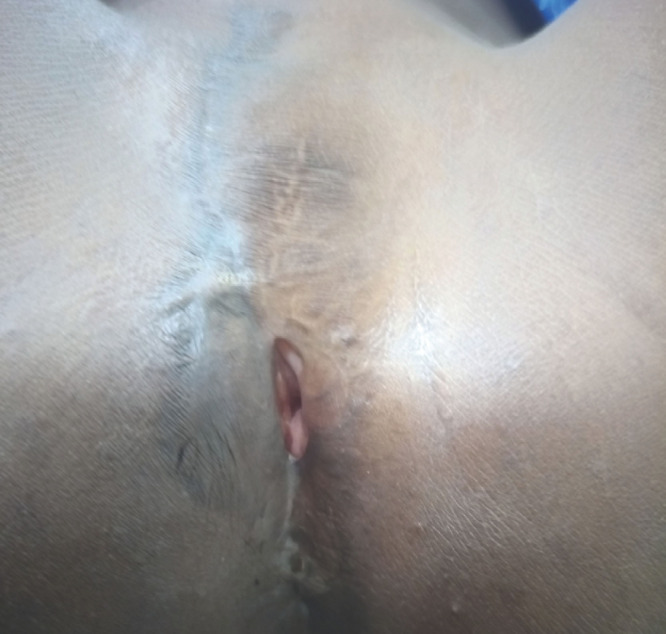
Aspect de la vulve au deuxième mois de la désinfibulation Appearance of the vulva in the second month of the deinfibulation

Une désinfibulation a été indiquée. Sous anesthésie générale, après asepsie loco-régionale, une ponction de l’épanchement intravaginal a été réalisée à l'aide d'une seringue, en vue d'un examen bactériologique. Puis, une incision verticale d'un centimètre de longueur jusque dans la cavité vaginale a été pratiquée sur la fibrose, à partir du pertuis. L'ouverture ainsi créée a été ensuite élargie aux ciseaux, en avant, jusqu'au repérage du méat urétral qui était intact, et en arrière. Une sonde urinaire à demeure a alors été placée, et un prélèvement d'urine réalisé pour la bactériologie. Les berges de l'incision ont ensuite été suturées sur tout le pourtour, en surjet avec du fil de suture résorbable 3/0, les points de suture reliant la peau et la muqueuse vaginale. La sonde urinaire a été gardée pendant 48 heures et des soins locaux consistant à garder de petites compresses enduites de néomycine au niveau du vestibule de la vulve ont été prescrits. *Escherichia coli* a été isolé des deux prélèvements, avec une sensibilité à la céfixime qui a été ajoutée aux soins post-opératoires. En définitive, le diagnostic d'infibulation compliquée de rétention chronique d'urine, d'urocolpos infecté et de reflux d'urine dans l'utérus a été retenu. Les suites opératoires ont été simples, marquées par la perméabilité de l'orifice vulvaire (Fig. 4), la disparition des signes urinaires et de l'urocolpos lors des visites de suivi du septième jour et des premier et deuxième mois. La cicatrisation complète a été constatée au premier mois.

### Cas clinique 2

Une fillette de 15 mois a été reçue en consultation pour des douleurs pelviennes et une dysurie de poussée. Les symptômes évoluaient de façon quasi permanente depuis 5 mois suite à une mutilation génitale rituelle. La patiente a été amenée plusieurs fois en consultation dans le centre de santé de son village où de l'amoxicilline et du paracétamol lui auraient été souvent prescrits. Longtemps ses parents auraient refusé sa référence au Centre hospitalier universitaire régional de Ouahigouya de peur d’être dénoncés à la police, pour avoir pratiqué une mutilation génitale.

**Figure 5:**
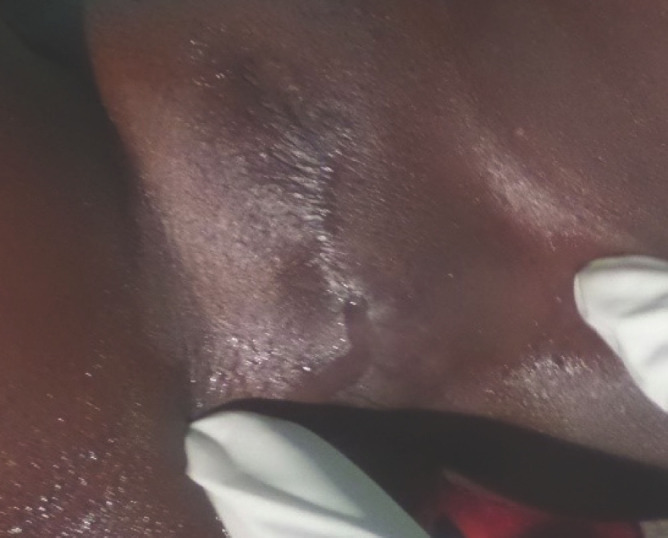
Infibulation avec accolement des grandes lèvres. Il est impossible de placer une sonde urinaire Infibulation with fusion of the labia majora. It is not possible to place a urinary catheter

L'examen clinique a retrouvé un état général conservé, une absence de syndrome infectieux, une légère voussure hypogastrique et une masse hypogastrique douloureuse, rénitente et mate. Au niveau de la vulve, il existait une cicatrice fibreuse d'une infibulation avec un accolement des grandes lèvres. Un petit pertuis au sein de la fibrose laissait s’écouler des urines à la poussée ou lorsque l'on exerçait une pression sur l'hypogastre ou sur la vulve. Il était impossible de placer une sonde urinaire (Fig. 5). Une sténose du méat urinaire compliquant une infibulation a été évoquée comme diagnostic.

**Figure 6:**
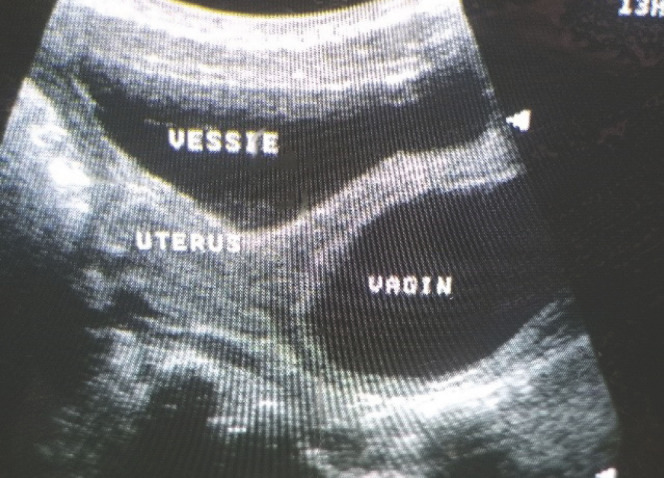
Image échographique montrant un urocolpos Ultrasound picture showing an urocolpos

L’échographie abdomino-pelvienne a été réalisée à l'admission, avant et après une miction. Elle a noté aux deux temps respectifs, des volumes urinaires de 58 ml et 32 ml. La cavité vaginale était distendue par un épanchement liquidien (Fig. 6). Il n'y avait pas de dilatation des cavités pyélo-calicielles. La créatininémie et l'hémogramme étaient normaux. Une ponction du liquide intravaginal a ramené des urines troubles dont l'examen bactériologique a permis d'isoler *Escherichia coli* sensible à l'imipenème. Cette ponction a été réalisée au début de la procédure de désinfibulation qui a été indiquée, juste après l'anesthésie générale. Ainsi, cette désinfibulation a permis de libérer le méat urétral qui était normal et d’évacuer le contenu du vagin. Le diagnostic d'une infibulation compliquée de rétention urinaire chronique et d'urocolpos infecté a été retenu. Une antibiothérapie avec de l'imipenème et des soins locaux à base de néomycine pommade ont été institués dans les suites opératoires qui ont été simples. La patiente revue une semaine, un mois puis deux mois après le traitement, n'a plus présenté ni dysurie, ni rétention d'urine, ni urocolpos. Son orifice vulvaire est resté perméable après la cicatrisation complète constatée lors de la deuxième visite.

## Discussion

### Épidémiologie

L'urocolpos et le reflux d'urine dans l'utérus qui peut lui être consécutif, sont des évènements rares, qui peuvent cependant se voir à tout âge, du fait de mécanismes différents [4]. Dans les deux cas que nous décrivons, les mutilations génitales de type infibulation étaient la cause de la fermeture du vestibule de la vulve par l'accolement des grandes lèvres, avec constitution d'une fibrose cicatricielle qui recouvrait le méat urétral. Au sein de la fibrose, il existait un pertuis permettant une miction difficile, et sous la fibrose, une communication entre le méat et le vagin. Cette communication entre le vagin et le méat était probablement plus large que le pertuis qui faisait communiquer le méat et l'extérieur à travers la fibrose de la cicatrice de l'infibulation. Ainsi, l'urine s’écoulait sous pression par le pertuis à l'extérieur, mais refluait également plus facilement dans le vagin où elle se collectait. Du vagin, l'urine refluait dans la cavité utérine pour l'un des cas que nous décrivons.

Les mutilations génitales féminines sont encore fréquentes dans le monde, malgré des décennies de lutte pour leur abandon [11]. Parmi les 4 types de mutilations génitales féminines de la classification de l'Organisation mondiale de la santé (OMS) [8], le type III qui est l'infibulation, est le plus grand pourvoyeur de complications [3, 8, 9]. Du fait de la fermeture de l'orifice vaginal par l'accolement des petites lèvres et/ou des grandes lèvres, il entraîne à moyen ou long terme, au niveau loco-régional, de nombreuses complications: un ralentissement de la menstruation et de la miction ainsi que des douleurs lors de celles-ci, un hématocolpos, une incontinence urinaire.

L'incontinence urinaire pourrait être une incontinence par regorgement du fait d'une rétention chronique d'urine, ou être due à la stagnation de l'urine sous le capuchon formé par les tissus cicatriciels [9]. Avec la possibilité d'urocolpos dans les suites d'infibulation que nous présentons, nous pouvons ajouter que l'incontinence urinaire pourrait également être due à un urocolpos dont elle est l'un des signes les plus constants [2, 4, 10]. L'absence d'incontinence urinaire pour nos deux cas est due au rétrécissement trop important du pertuis permettant l'extériorisation des urines. Ganta et al. ont d'ailleurs cité les mutilations génitales comme une cause d'urocolpos [4].

Dans la littérature, le mécanisme le plus souvent décrit dans la genèse de l'urocolpos est la coalescence des petites lèvres due à diverses lésions de la vulve, dans un contexte d'hypo-œstrogénie [2, 4]. Les causes malformatives congénitales sont également décrites: l'hypospadias, la fusion labiale [7]. Enfin des causes fonctionnelles de reflux vésico-vaginal d'urines ont été rapportées: il s'agit de cas survenant lorsque le flux urinaire rencontre un obstacle dans les grandes lèvres, généralement lorsque les membres pelviens ne sont pas suffisamment en abduction lors de la miction [5]. Lorsque la pression dans le vagin augmente du fait d'un épanchement important d'urine, il se produit un reflux dans la cavité utérine. À partir de l'utérus, ce reflux peut se faire vers les trompes, et être une cause de péritonite [1, 2].

### Diagnostic

Les signes cliniques des patientes que nous présentons n'ont pas fait évoquer le diagnostic d'urocolpos. Pourtant, certains éléments auraient pu orienter vers ce diagnostic: il s'agit de la vulve rénitente et de l’écoulement d'urines par le pertuis au sein de la fibrose lors de la pression de la vulve. Dans la littérature, le signe clinique le plus constant en cas de reflux d'urine dans le vagin et d'urocolpos est l'incontinence urinaire post-mictionnelle [2,4,5,10]. Pour nos deux patientes, du fait du petit calibre du pertuis permettant d’évacuer les urines lors de la miction, ce signe clinique ne pouvait être objectivé. Ganta [4] a noté également qu'une distension vaginale au toucher rectal pourrait être un signe d'urocolpos. Ce signe n'a pas été recherché chez nos patientes.

L’échographie a été essentielle pour nous dans le diagnostic, mais aussi pour plusieurs auteurs [4, 10]. De même la cysto-urétrographie permet de mettre en évidence le reflux intravaginal d'urine permictionnel et la constitution d'un urocolpos [5]. Mais cet examen ne pouvait être réalisé chez nos patientes du fait de l'impossibilité de cathétériser la vessie.

Grâce à la bactériologie, nous avons identifié une bactériurie à *Escherichia coli* chez les deux patientes dont nous décrivons les cas. La proximité entre les voies digestives et urinaires pourrait expliquer l'isolement de ce germe qui est une entérobactérie. La pullulation bactérienne, quant à elle, est favorisée par la stase urinaire [5].

### Traitement

Une désinfibulation, une antibiothérapie et des soins locaux ont été nécessaires pour la prise en charge. Ces soins étaient justifiés pour restaurer une miction normale et la perméabilité de la vulve et du vagin [8]. À court, moyen et long termes, le vestibule de la vulve est resté ouvert, et la miction est devenue normale. La possibilité de fermeture de l'orifice vulvaire après une désinfibulation est plutôt le fait de procédures délibérées de réinfibulation [8]. Cependant, d'autres types de complications liées aux mutilations génitales sont à craindre pour nos patientes. Ce sont les risques psychologiques à moyen ou long termes, et plus tard à l'adolescence et à l’âge adulte, les risques obstétricaux, et les dysfonctionnements sexuels [9].

## Conclusion

Les mutilations génitales féminines sont des causes rarement décrites d'urocolpos avec reflux dans la cavité utérine. Elles sont pourtant encore fréquentes, même dans leur forme la plus mutilante qu'est l'infibulation. Cela suggère que l'urocolpos pourrait être plus fréquent, mais peu diagnostiqué. Au vu des cas que nous présentons et de la revue de la littérature, les praticiens devraient penser à la possibilité d'un urocolpos, en cas d'infibulation, devant une incontinence urinaire permanente, une vulve rénitente laissant s’écouler des urines à la pression, et un vagin bombé au toucher rectal. La cysto-urétrographie, lorsqu'elle est possible, permet de mettre en évidence le reflux urétro-vaginal d'urine, et l’échographie pelvienne de poser le diagnostic de l'urocolpos. Ce dernier est potentiellement grave car pouvant s'infecter, et provoquer une péritonite. Sa prise en charge adéquate est alors nécessaire, et surtout, il est urgent d'accentuer la lutte pour l'abandon de la pratique des mutilations génitales féminines.

## Principes éthiques

Les parents des patientes ont donné leur accord pour la réalisation des photographies, et ont également consenti à leur utilisation dans cette publication.

La direction des services médicaux et techniques du Centre hospitalier régional de Ouahigouya qui a en charge les questions éthiques a donné son accord pour cette publication.

## Lien d'intérêts

Les auteurs ne déclarent aucun lien d'intérêt.

## Contribution des auteurs

Sansan Rodrigue SIB, Moussa SANOGO et Issa OUEDRAOGO ont participé à la prise charge des patientes. Ils ont rédigé la première version du manuscrit.

Evelyne KOMBOIGO, Sibraogo KIEMTORE, et Ali OUEDRAOGO ont apporté des contributions essentielles au document.

Sansan Rodrigue SIB et Evelyne KOMBOIGO ont coordonné la rédaction du manuscrit.

Tous les auteurs ont lu et approuvé le document final.

## References

[1] Beale G, Hackett AH (1969). A case of uterine reflux of urine in a girl and its suggested role in primary pneumococcal peritonitis. N Z Med J.

[2] Centeno-Wolf N, Chardot C, Le Coultre CP, La Scala GC (2008). Infected urocolpos and generalized peritonitis secondary to labia minora adhesions. J Pediatr Surg.

[3] Collinet P, Sabban F, Lucot J-P, Boukerrou M, Stien L, Leroy J-L (2004). Prise en charge des mutilations génitales féminines de type III. J Gynecol Obstet Biol Reprod.

[4] Ganta SJ, Kulkarni S, Thomas N (2017). A rare case of urocolpos. Int J Reprod Contracept Ostet Gynecol.

[5] Haouas N, Cariou G (2006). Le reflux urétro-vaginal permictionnel. J Gynecol Obstet Biol Reprod.

[6] Leung AK, Robson WL, Kao CP, Liu EK, Fong JH (2005). Treatment of labial fusion with topical estrogen therapy. Clin Pediatr (Phila).

[7] Mikhel'son AI, Trofimova ZA (1970). [Urocolpos in vaginal ectopia of the ureter of a hypoplastic kidney in an 8-month-old infant]. Urol Nefrol (Moscou).

[8] OMS (2018). Lignes directrices de l'OMS sur la prise en charge des complications des mutilations sexuelles féminines [WHO guidelines on the management of health complications from female genital mutilation].

[9] OMS Complications sanitaires des mutilations sexuelles féminines.

[10] Ruiz RM (2015). Reflujo vaginal, una causa frecuente de incontinencia urinaria [Vaginal reflux: A common cause of urinary incontinence]. An Pediatr (Barc).

[11] Yoder PS, Shanxiao Wang Female genital cutting: the interpretation of recent DHS data. DHS Comparative Reports No. 33.

